# Exploring Attitudes and Beliefs towards Implementing Cattle Disease Prevention and Control Measures: A Qualitative Study with Dairy Farmers in Great Britain

**DOI:** 10.3390/ani6100061

**Published:** 2016-10-11

**Authors:** Marnie L. Brennan, Nick Wright, Wendela Wapenaar, Susanne Jarratt, Pru Hobson-West, Imogen F. Richens, Jasmeet Kaler, Heather Buchanan, Jonathan N. Huxley, Heather M. O’Connor

**Affiliations:** 1 School of Veterinary Medicine and Science, The University of Nottingham, Sutton Bonington Campus, Loughborough LE12 5RD, UK; wendela.wapenaar@nottingham.ac.uk (W.W.); pru.hobson-west@nottingham.ac.uk (P.H.-W.); imogen.richens@nottingham.ac.uk (I.F.R.); jasmeet.kaler@nottingham.ac.uk (J.K.); jon.huxley@nottingham.ac.uk (J.N.H.); heather.m.oconnor@outlook.com (H.M.O.); 2 The University of Nottingham, University Park Campus, Nottingham NG7 2RD, UK; nick.wright@nottingham.ac.uk (N.W.); susanne.jarratt@talk21.com (S.J.); 3 Queen’s Medical Centre, The University of Nottingham, Nottingham NG7 2UH, UK; heather.buchanan@nottingham.ac.uk

**Keywords:** health psychology models, theory of planned behaviour, biosecurity, disease prevention, disease control, cattle farmer/s, dairy, attitude, belief

## Abstract

**Simple Summary:**

Further understanding of why dairy farmers do not engage in disease prevention and control strategies (biosecurity) is required. Using semi-structured interviews informed by a health psychology approach with 25 dairy farmers, a number of barriers, such as disease testing inaccuracies, types of disease transmission, perceived lack of risk and effectiveness of measures, were identified. Motivators included being advised to undertake measures by veterinarians, and the increased threat and severity of the disease in focus. These results suggest there is an advantage to farm advisors and herd health professionals understanding and working with the beliefs of individual dairy farmers to target appropriate communication and advice strategies relating to biosecurity recommendations.

**Abstract:**

Disease prevention and control practices are frequently highlighted as important to ensure the health and welfare of farmed animals, although little is known as to why not many practices are carried out. The aim of this study was to identify the motivators and barriers of dairy cattle farmers towards the use of biosecurity measures on dairy farms using a health psychology approach. Twenty-five farmers on 24 farms in Great Britain (GB) were interviewed using the Theory of Planned Behaviour framework. Results indicated that farmers perceived they had the ability to control what happened on their farms in terms of preventing and controlling disease, and described benefits from being proactive and vigilant. However, barriers were cited in relation to testing inaccuracies, effectiveness and time-efficiency of practices, and disease transmission route (e.g., airborne transmission). Farmers reported they were positively influenced by veterinarians and negatively influenced by the government (Department for Environment Food & Rural Affairs (DEFRA)) and the general public. Decisions to implement practices were influenced by the perceived severity of the disease in question, if disease was diagnosed on the farm already, or was occurring on other farms. Farmers described undertaking a form of personal risk assessment when deciding if practices were worth doing, which did not always involve building in disease specific factors or opinions from veterinarians or other advisors. These results indicate that further guidance about the intricacies of control and prevention principles in relation to specific animal diseases may be required, with an obvious role for veterinarians. There appears to be an opportunity for farm advisors and herd health professionals to further understand farmer beliefs behind certain attitudes and target communication and advice accordingly to further enhance dairy cattle health and welfare.

## 1. Introduction

It has long been recognised that the prevention and control of diseases, sometimes known by the term biosecurity, on animal production enterprises can result in a number of positive benefits for animal health and welfare [1,2,3,4]. Much work has previously been carried out by researchers looking at the biosecurity measures, including vaccination [5], that are or are not employed by farmers in a number of countries across a variety of animal businesses, including cattle operations [6,7] and pig, sheep and goat operations [8,9]. Often, it has been reported that very few practices are being undertaken, particularly by cattle farmers. More recently, the role of other stakeholders, including veterinarians, in on-farm biosecurity has also been explored [10,11,12].

Questions about why measures are not undertaken more by farmers have been addressed in the literature. A number of studies have focused on what farmers think and feel about these preventive practices to try and identify what approaches or resources would assist in optimising farmer uptake of preventive measures [13,14,15,16]. Without the use of theoretically robust and empirically validated behaviour change frameworks, it can be difficult to determine how effective any approaches designed to encourage altered behaviour will be [17]. Health psychology theories can identify underlying beliefs which form attitudes and motivations and can relate an action to these beliefs [18,19,20]. These theories help to potentially explain which factors are most influential on a given health behaviour and how these factors interact with each other [21,22]. Some of these theories have been utilised previously within the agricultural sphere and are listed in Table 1; this list is illustrative and not exhaustive. 

**Figure 1:**
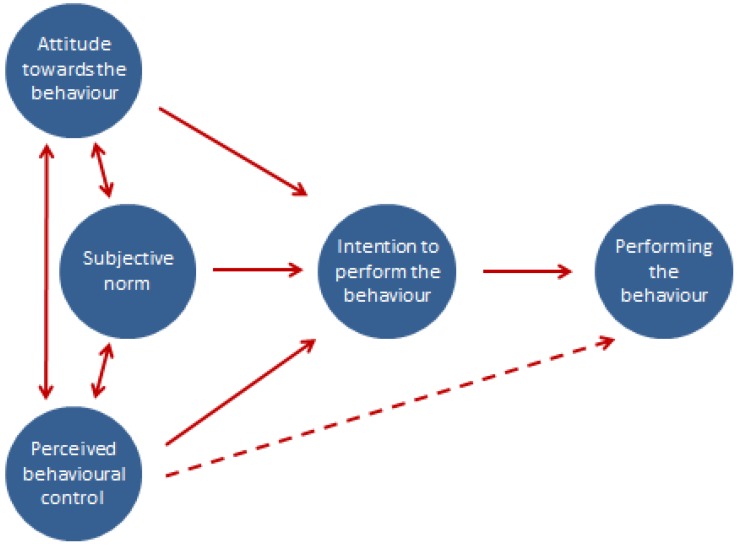
Schematic representation of the Theory of Planned Behaviour. Adapted from [34].

One of the most frequently applied models used within the health psychology field is the Theory of Planned Behaviour [33]. The Theory of Planned Behaviour (TPB) aims to determine the association between attitudes, beliefs, intentions and the perceived behavioural control an individual feels they have over a particular behaviour, as well as how these perceptions predict actual behaviour; Figure 1 [20,33]. 

Broadly, the three main components in this theory are:

- Behavioural belief (Attitude towards the behaviour)—The belief that a behaviour leads to a certain outcome, for example, a farmer feels that by implementing biosecurity measures, the productivity of their cattle will subsequently improve.
- Normative belief (Subjective norm)—The belief that particular individuals or groups think a person should or should not perform the behaviour, for example, a farmer believes his/her milk buyer thinks it is important that he/she implements biosecurity measures on his/her farm.
- Control belief (Perceived behavioural control)—Someone’s perception of their own ability to perform a behaviour, for example, how able a farmer feels they are to prevent diseases coming onto their farm.

This model has been successfully applied to the development of behaviour change programmes for human health-associated behaviours, such as hand hygiene practices and sun protection measures [35,36,37] and, similarly, animal health-associated behaviours around conditions such as bovine mastitis [38]. Potentially, the TPB model could be utilised to assess what the most important barriers and motivators are to farmers undertaking biosecurity practices in order for targeted programmes to be developed to improve uptake of measures and subsequently enhance animal health and welfare.

Therefore, the aim of this study was to use interviews structured using the TPB to identify the ways in which dairy farmers in Great Britain experience motivators and barriers to implementing biosecurity measures.

## 2. Materials and Methods

### 2.1. Population

The population of interest was dairy cattle farmers in Great Britain (GB). The sampling frame used to identify potential participants was the Agriculture & Horticulture Development Board (AHDB) Dairy database (AHDB Dairy is a levy funded, not-for-profit organisation working on behalf of British dairy farmers). Audio-recorded semi-structured telephone interviews were used to collect the data. 

### 2.2. Participant Selection

Farmers were selected accounting for factors that were likely to affect attitudes towards biosecurity (geographical region, herd size, and type of enterprise, e.g., conventional or organic). The purpose of the participant selection method was to use a maximum variation sampling approach [39]. To do this, GB was split into six geographical regions according to DEFRA regional office areas [40]. Farms were then sub-divided into four herd sizes (small = 1–49 cattle; medium = 50–149 cattle; large = 150 cattle and over; unknown = unrecorded; DairyCo., Kenilworth, UK, 2012), and also by type of enterprise (organic and conventional). A selection of farmers was then taken from the database and sent by AHDB Dairy to the researchers. Within each herd size category, farmers were randomly sorted prior to recruitment. The aim was to recruit one small conventional, one medium conventional, one large conventional and one organic (of any herd size) farmer per region, leading to a total of 24 farmers being interviewed. 

### 2.3. Farmer Recruitment

Farmers’ addresses within the AHDB Dairy database were used to search for phone numbers via The Phone Book (www.thephonebook.bt.com) and Yell (www.yell.com). If phone numbers could not be sourced for farmers selected in the list, farmers were sent a letter asking if they wanted to participate in the study, and it included a return form identifying if they were willing to participate or not. A self-addressed envelope was included to facilitate the process. An incentive prize draw (£100 voucher for a business of their choice) was offered.

Farmers were telephoned in order from the top of each randomly sorted herd size category within each region. The herd size was double checked with the farmer. Farmers who did not answer the phone, or where an answerphone was encountered, were tried multiple times (no message was left on answerphones). This process was carried out until a farmer in a particular region, of a particular herd size agreed to participate. The conventional farmers were recruited and interviewed first followed by the organic farmers.

### 2.4. Farmer Interviews

Farmers willing to participate were interviewed via telephone by an experienced female postdoctoral researcher (Susanne Jarrett) at the most convenient time for the farmer, and all interviews were audio-recorded. At the beginning of each interview, consent was given verbally by the farmer to participate in the interview, and permission was given for the interview to be recorded. The interviews were designed to follow a semi-structured approach and were conducted using the same interview guide (questions and wording) for all interviewees (Appendix A). A pilot study involving five farmers was conducted to ensure that the interview guide worked in the desired manner and that the recording mechanism was functional; small changes were made to the interview guide at this stage after discussion amongst three authors (Marnie Louise Brennan, Susanne Jarrett, Heather Marie O’Connor). 

Participants were firstly asked general questions about themselves and their farm, such as how long they had been farming, the number of dairy cattle on the farm, and whether any other production systems were run. They were then asked to describe what they felt posed the biggest threat to their farm, and what disease on a dairy farm meant to them. Questions were then asked based on the constructs of the TPB (three main questions). These questions centred on the three belief components of this model; behavioural beliefs, normative beliefs, and control beliefs. These three questions were structured using the expression “disease prevention and control measures”. This was followed by asking the same three key questions but in relation to using the term “biosecurity”. The different terminologies were used to ensure that any misconceptions about the word “biosecurity” would be minimised. Each question had a number of prompt or follow-on questions that could be used if further exploration was required.

### 2.5. Data Analysis

Interviews were transcribed verbatim by a commercial transcriber. All interview transcripts were read by the researcher who interviewed the farmers (Susanne Jarrett) and checked with the recordings to ensure accurate transcriptions. Transcriptions were then anonymised and imported into analysis software (Nvivo 10, QSR international, London, UK).

Thematic analysis was used to analyse the data [41,42]. Thematic analysis is a method for identifying, analysing, and reporting patterns (themes) within data and has been successfully applied in other veterinary studies [43]. A proportion of the interviews (*n* = 5; 21%) were coded independently by two researchers (Marnie Louise Brennan and Susanne Jarrett) prior to in-depth analysis. A comparison was made between the two code lists created, and small adjustments were made before a single researcher (Susanne Jarrett) coded the remaining interviews and re-coded the initial five interviews. The TPB provided an initial coding framework, with text associated with each formal TPB question initially coded against a node representing the formal question (a form of inductive analysis). If responses relevant to any of the three TPB constructs were identified in other areas of the questionnaire, the response was assigned to the relevant TPB node. After this process, other codes were created based on themes and topics coming from the data which were additional to the TPB concepts. Additionally, after the inductive analysis had been undertaken, a second researcher (Marnie Louise Brennan) performed a form of deductive analysis on the themes identified from the analysis to reduce down the smaller themes into “global” themes [41].

### 2.6. Ethical Consideration

The study received ethical approval from the School of Veterinary Medicine and Science Ethics committee at The University of Nottingham. 

In the results section below, individual participants are depicted by an abbreviation at the end of each quote (e.g., Farmer 2 would be F2) to ensure anonymity and protection of participants. Direct quotes are used to exemplify themes in the data.

## 3. Results

Twenty-five farmers from 24 farms were interviewed in total (excluding the five interviewed during the pilot study; Table 2). A joint interview was conducted with two farmers (mother and son) from the large South West conventional farm. A farmer with a small herd in the South East region who was willing to participate could not be identified, so a replacement farmer with a large herd was interviewed; no small organic farmers were identified.

### 3.1. General Findings

Most farmers believed that disease was likely on farms and that the majority felt it was worth taking actions to prevent or control disease.

“I think it’s quite highly likely to get disease in. Especially if you’re buying in stock, which I’m not doing. But dairy farms have an awful lot of visitors and different people coming through, which could potentially be possibly carrying or unknowingly carrying disease. It just takes your neighbours who have got the disease to go and put something in your field. I don’t think we’re immune to it, no.”

(F10)

However, a proportion of farmers were unsure or thought their animals were unlikely to get disease, regardless of whether the herd was open or closed.

### 3.2. Behavioural Beliefs

Being proactive and vigilant on farms was considered beneficial by many farmers, particularly those with closed herds. Proactive testing and monitoring was deemed helpful in identifying problems earlier and enabled the farmers to deal with problems more quickly.

“We have, we are very strict about keeping a close monitoring (of) them, a close eye on the animals so that if there is something that goes amiss that you are there at the beginning rather than you know towards the disastrous stage.”

(F7)

“It’s just about being conscientious about your animals’ health status and trying to protect it at all costs.”

(F3)

However, the alternative argument was also made in relation to having disease on farms resulting in “protective exposure”.

F6’s experience with the introduction of digital dermatitis into a closed herd: “…there’s a certain amount of resistance that definitely develops with the cows we have now because they have had it all the way through…”

Farmers chose to undertake (or not) measures based on a risk judgement made by them, in relation to their perceived risk or likelihood of getting disease.

“But, you know, I could spend hundreds of pounds on chemicals to try and keep everything clean, and spend hours and hours keeping it all clean, but at the end of the day the chances are we’re not likely to get that disease anyway, or whatever. Some of the big ones only come every once in a blue moon hopefully.”

(F8)

Additionally, some farmers felt that it was difficult to engage in preventive approaches when inaccuracies in disease control programmes existed.

“And quite a lot of those cows are actually slaughtered unnecessarily because the test (for bovine tuberculosis) in cattle is not very reliable.”

(F13)

There was also a feeling that regardless of the actions carried out on a farm, there were occasions when preventive measures were not going to be effective or time efficient.

“…Because you can put a lot of effort into it and give yourself ten out of ten for what you’re doing…There’s a lot of backdoor ways or other routes into the farm for disease problems to come that are beyond your control.”

(F4)

“…because all of these things are alright in isolation but when we are not really being paid sufficiently for our product to cover all of the time that they then spend, then you know you have to look at that and think well actually you know is it more important to do that or to go in and monitor the cows that are calving in the next field. You have to make day to day assessments of where your time is best spent.”

(F7)

### 3.3. Normative Beliefs

There were only a few influencers farmers nominated as potentially having a role to play in relation to biosecurity practices on farms. Not surprisingly, veterinarians were mentioned by the majority of farmers.

“Er, I wanted to introduce more Jersey into the herd, the vet said breed it in yourself, whatever you do, don’t buy anything in because your herd health status is so high, don’t risk buying in any other diseases.”

(F4)

“Well, at the moment it’s just a closed herd, so we’re obviously not bringing animals in. And then if there was a threat of a disease from a neighbouring farm I would speak to the vet and take his advice and go from there.”

(F9)

DEFRA were also frequently mentioned as having a role to play in providing guidelines or advice about biosecurity, but the guidance was not always seen as effective or relevant.

“Well, DEFRA bring out some quite extraordinary rules but they don’t seem to want…they don’t seem to work. They don’t seem to be practical enough. It’s just, a lot of farmers think it’s just to keep people in work thinking up the next rule and regulation.”

(F20)

“I mean I suppose DEFRA would like to think it is sort of them but they often don’t make a very good job of it…”

(F19)

Interestingly, farmers felt that the public perceived they had a role to play in how farming should operate and the practices undertaken on farms, which they felt were not always informed or welcomed.

“Well, in general, I think the public want you to use as little medicine as possible. I think—and they’re probably…generally…they want you to use preventative measures, probably want you to use preventative measures than actual using medicines, I’d say that would be.”

(F11)

“Well I think lots of people have got opinions, probably agriculture suffers, more from other people’s perceptions and their opinions derived from those perceptions than any other industry. I mean we all have opinions on the health service and the police service and all the rest of it. But I think because we are, we’ve come from hunter gatherers everybody feels they have got the right to have an opinion on the land, on the way we produce our products from our animals. And I have got no problem with that except for a lot of it is you know a little bit of information is dangerous.”

(F7)

### 3.4. Control Beliefs

Many farmers felt like they possessed the ability to control what happened in their herd in relation to combating disease using preventive biosecurity measures.

“I just take a decision on a daily/monthly/yearly basis which may or may not vary, for instance things altering, there’s a foot and mouth outbreak obviously we shut the farm gate and we let virtually no one on.”

(F23)

“If it comes to the push, yes, just shut the gates and stop anybody coming in.”

(F2)

However, there were circumstances where this ability to control was perceived as difficult. These difficulties seem to be based around types of diseases, such as those perceived to be transmitted by wildlife or were airborne.

“I think really like highly infectious airborne diseases I have got no control over. You know if we get foot and mouth come through, I mean short of putting snipers on the farm borders and shooting everything that crosses across including birds and everything else, you know I mean prevent everything…”

(F6)

“If you’re trying to, it’s very hard to try and keep TB out, unless you fence all your farm with ten feet high fences so that the deer can’t come over, dig it ten feet deep in the ground so the badgers can’t bore underneath it and that’s the only way you can stop it.”

(F20)

“I’ve had that (Schmallenberg). There’s nothing I can do about that, I don’t think. If it’s midges flying, you know, across the Channel or whatever, and they attack my cows, that’s the way it is, there’s not much I can do with that. So no, I don’t think I can do much for that sort of thing.”

(F8)

Additionally, there was a feeling that there were only so many actions that farmers could take, which was not unlimited because of other tasks or responsibilities. This led to a feeling that you could only do “so much”.

“Well, we do it, we’re doing our best but um, we can only do so much.”

(F20)

“You know, you can only, you can only do so much, you can only do foot bathing and, um, well, nobody really has got the time to wash themselves down with disinfectant.”

(F1)

### 3.5. Specific Factors Encouraging Farmers to Implement Biosecurity Measures

There were a variety of factors highlighted by farmers as being likely to encourage them to undertake biosecurity measures. The type and severity of disease that the farm, region or country was currently under threat from was one of the main factors mentioned. This was particularly in relation to foot and mouth disease.

“…If there’s serious contagious disease in or near the farm or not in the farm, in or near, in the country or near the farm then that’s what we would do.”

(F23)

“…so we try to keep it away from other cattle. We don’t do anything else I don’t think, too much. The disinfecting side, it sort of was very popular when we were—with Foot and Mouth—but after that it seems to have died a death now.”

(F24)

A number of farmers extended this, stating that if the disease moved from being just a threat to having disease on their farm, or if a disease outbreak was occurring, they would be more likely to undertake measures.

“Well, we have them tested to see what we’ve got. And then we, you know, go by that, rather than just injection willy-nilly for this that and the other. There’s no point trying to prevent something they haven’t got is it?”

(F18)

“Biosecurity on a farm is, it’s a bit of a, I don’t know, it’s kind of a tricky thing because we all know what we should do but what actually gets done is very little until you have actually a problem…but if there was an outbreak I’d have a foot dipper, a wheel wash up. So biosecurity is kind of, it’s doing the least possible when necessary.”

(F6)

A number of farmers appeared to be very much guided by what their veterinarian advised them to do in relation to whether to undertake a measure or not.

“And then if there was a threat of a disease from a neighbouring farm I would speak to the vet and take his advice and go from there.”

(F9)

Farmers felt that if there was proof demonstrating that the preventive practices actually worked they may be more likely to undertake them. Additionally, it appeared that preventive measures were more likely to be undertaken by farmers if they perceived the benefits outweighed the costs.

“If it worked, if it was shown to work. It’s no good making yourself do more work than you have to is there. Farming’s hard enough.”

(F20)

“What would make (you take up a new biosecurity measure)—well, if it was guaranteed to do something I’d perhaps do it. If it was feasible and economical and not too difficult. But feasibility and economical would be the best bet. If I knew it was—if I knew it cost me five pounds to implement something and took me no time at all to do it, and it was earning me ten quid, then you’re quite happy, aren’t you, really?”

(F8)

## 4. Discussion

Using a health psychology approach, with the TPB framework, has enabled a classification of motivators and barriers which farmers identified as important influences as to whether they undertook disease prevention and control procedures on dairy cattle farms. This insight enables a deeper understanding of what additional strategic approaches are required to further assist farmers and other industry players to adopt additional measures to protect the health and welfare of dairy cattle by preventing disease occurring on farms. It is unlikely this rich data, collected by in-depth interviews, could be gathered via more traditional quantitative methods such as questionnaires. This sort of approach could be useful for current disease control initiatives, such as the Scottish Bovine Viral Diarrhoea (BVD) Eradication scheme [44] and TB hub [45] strategies, and those in the future. 

This study has identified that some farmers feel they have the ability to affect whether or not they get diseases on farms, although there are some perceived barriers to this relating to the understanding of how diseases are transmitted and, perhaps, the likelihood of transmission routes, such as diseases relating to wildlife and airborne diseases. This perceived ability to influence whether diseases affect animal herds has been found in a similar study conducted in Sweden [4]. The idea of exposing cattle to disease as a “protective” measure is an interesting finding and has been recognised as something seen more frequently in cattle farmers than in farmers of other species [4]. Depending on the specific disease, this idea of “protecting” animals by previous disease exposure could have severe consequences on farmers’ production. This suggests that those advising farmers about disease preventive practices should not necessarily presume farmers have an understanding of the short or long term consequences of getting a disease on their farm and should be aware of the existence of the farmer theory of “preventive exposure”. It is possible that this theory is as a result of historical advice relating to previous understanding of disease processes that conflict with more recent research evidence. An example is BVD virus and the traditional view of persistently infected (PI) animals acting to “vaccinate” the remainder of the herd [46]. 

It is apparent that some dairy cattle farmers may be making judgements about whether preventive practices are worth undertaking based on their own individual risk assessment which is likely to be informed by previous experience and tacit knowledge, which has been reported in a recent study [33]. It has been shown that risk can mean different things to different people, with experts and lay people contrasting in their perception of risk of the same activity [47]. Risk and trust have been seen to be closely intertwined, particularly in relation to human vaccination [48]. Farmers may be more likely to rely on their own perceptions of risk if they do not trust the sources of advice on preventive practices [49]. There is a perception by some farmers in the current study that an “all or nothing” approach is required, relating to the perceived constraints on time or effort to perform a lot of the measures, particularly in relation to diseases such as foot and mouth disease or bovine tuberculosis. Additionally, the idea of having to deal with uncertainties such as inaccurate disease testing and a lack of proof that practices are effective are also perceived barriers. This lack of proof of efficacy, and disease transmission likely to occur despite effort, has been suggested in previous studies and other production species [15,31,50]. Animal health advisors clearly have a role to play, specifically veterinarians, as they are perceived as trusted and are relied upon for advice on combating disease [14,51,52], although not always utilised [13,53,54]. The potential role of DEFRA in promoting biosecurity, although acknowledged by farmers, appears unlikely to lead to successful change considering the findings here, and those found previously [47]. Veterinarians may need to take a more proactive approach in relation to identifying what factors have been considered during the farmers’ risk assessment, and advise accordingly as the likely experts in pathogen epidemiology, which may not be the farmers’ area of expertise. It has been suggested previously that discussion between veterinarians and farmers might lead to a larger number of practices being undertaken [16,51,54]. An awareness of farmers’ beliefs are required to prioritise interventions; if the recommendations are congruent with farmer beliefs, particular strategies are likely to be successful, and others counterproductive [55]. Additionally, there is a need for advisors to focus on communication around explaining the effect of uncertainties and the best course of action accounting for these ambiguities [56].

Farmers appear to be motivated to uptake measures after diseases have already been found on farms, specifically those perceived as having severe consequences or when there is a threat of a disease outbreak. This finding could be a result of the way the questions were asked in the interviews; terminology relating to disease prevention and control was used in the interview guide. It has been identified that biosecurity practices recommended during disease outbreaks may not be perceived as effective, or may result in negative connotations [13,57]. Furthermore, if people feel positive towards an approach they will judge it to have high benefit and therefore low risk, which is reversed if they feel negative about it [58]. Biosecurity practices can be used as part of both disease prevention and control strategies, and it may be useful for advisors to clarify with farmers which role their recommendations relate to when discussing approaches.

The results from this project were not designed to be generalised to all farmers in GB, but to explore the range of perceived barriers and motivators to undertaking disease prevention and control practices. Understanding of perceptions of disease can only be explored in-depth using qualitative research methods such as interviews [39] and can add significant value to quantitative approaches which are frequently used in animal health research. Participants may have been more likely to respond to questions in a particular way for fear of not answering as expected; to minimise this social acceptability bias, similar questions were asked in different ways, and during the interviews the farmers appeared consistent in their responses. A general critique of the application of qualitative research methods, such as interviews, assumes that individuals can actually provide accounts of their own beliefs. In reality, interviews are accounts of beliefs rather than methods by which concepts like beliefs can be measured [39]. Further work is being undertaken investigating how the results found here translate to a wider spectrum of dairy farmers and how prevalent these beliefs are.

There has been debate within the scientific literature as to the value of behaviour change approaches and whether attitudes are linked to actual behaviours [17]. Additionally, one of the key assumptions of the Theory of Planned Behaviour is that decision-making is “rational” [34]. However, it is likely that emotional and contextual factors [59] play a role in influencing behaviours and may not be accounted for when applying these approaches. There are other elements, such as pathogen transmission probability, the organization of farms and industry sectors, and the level of control and protection required to reduce risk that are likely to have a role to play in terms of control and prevention of disease [60].

Further exploration of this type of research and its relationship with effective translation to change should be considered.

## 5. Conclusions

The use of interviews and health psychology frameworks can help to further understand the complexity around decision-making in relation to the utilisation of preventive practices on dairy cattle farms in GB. This could be of benefit to disease control initiatives, and failing to do in-depth research of this type risks misunderstanding of how policies are experienced on the ground, ultimately affecting the likelihood of the success of these policies. Further research looking at how this work translates to a wider range of individuals and the role of the veterinarian and other industry stakeholders is planned to determine the best approach to disease prevention and control frameworks on farms.

## Figures and Tables

**Table 1:** An illustrative list of studies that have employed health psychology models in an agricultural setting.

Author and Year Published	Citation	Subject	Population	Health Psychology Model Used
Garforth et al., 2004	[23]	Knowledge and technologies transfer strategies	Cattle and sheep farmers	TRA
Ellis-Iversen et al., 2010	[24]	Zoonotic disease control	Cattle farmers	TPB/SEM
Valeeva et al., 2011	[25]	Risk of animal disease and risk management strategies	Pig farmers	HBM
Lind et al., 2012	[26]	Mastitis	Cattle farmers	TPB
Delgado et al., 2012	[27]	Foot and mouth disease detection and control	Cattle farmers	TPB
Bruijnis et al., 2013	[28]	Foot health	Cattle farmers	TPB
Espetvedt et al., 2013	[29]	Contact with veterinarian (mastitis)	Cattle farmers	TPB
Garforth et al., 2013	[15]	Disease risk management	Sheep and pig farmers	TRA/TPB/HBM
Jaaskelainen et al., 2014	[30]	Animal welfare and production; farmer disposition	Pig farmers	TPB
Alarcon et al., 2014	[31]	Disease control	Pig farmers	TPB
Delgado et al., 2014	[32]	Movement ban compliance during FMD control	Cattle farmers	TPB
Shortall et al., 2016	[33]	Biosecurity	Cattle farmers	SEM

HBM = Health Behaviour Model; SEM = Socioecological Model; TRA = Theory of Reasoned Action; TPB = Theory of Planned Behaviour.

**Table 2:** Dairy farmers interviewed to investigate attitudes towards disease prevention and control measures according to their location, herd size, and type of enterprise (organic versus conventional).

Herd Size and Type	Scotland	Wales	South West	South East	Midlands	North
Small (Conventional)	1	1	1	0	1	1
Medium (Conventional)	1	1	1	1	1	1
Large (Conventional)	1	1	1	2	1	1
Organic	Medium	Medium	Large	Large	Medium	Medium

## Appendix A. Interview Guide for the Farmer Interviews

*Q1. Tell me about your farm*

Prompts:

Whereabouts is your farm?

How long have you been farming?

How long have you had dairy cattle?

How many dairy cows do you have?

Other than dairy cattle, do you have any other production systems on your farm?

What is your set-up like there? Are you operating under any specific types of production?

Are you an open or closed herd? Do you use AI? Do you have your own bull?

Who is your buyer? Who are you supplying to?

Are you a member of any farm assurance schemes?

*Q2. What poses the biggest threat to your farm?*

*Q3. What does disease on a dairy farm mean to you?*

Prompts:

How likely is a dairy farm to get diseases?

How does disease get onto a dairy farm?

Where does disease come from?

*Q4. If I say disease preventions and controls, what does it make you think of?*

Prompts:

Is there something that you picture when someone says disease preventions and controls?

What is the word that comes into your head when you hear “disease preventions” and “disease controls”?

How do you feel when someone starts talking about disease preventions and control?

*Q5. What do other people think you should be doing to prevent or control diseases moving to and from your farm?*

Prompts:

If I say disease preventions and controls, who are the people you think of?

Who has an opinion on what you should be doing? What is that opinion?

Why don’t you just do what other people say?

*Q6. Are disease preventions and controls something you think you are really able to do?*

Prompts:

Can you prevent or control disease moving onto your farm?

Can you prevent or control disease moving off of your farm?

*Q7. Is taking actions to prevent or control disease moving to and from your farm really worth it?*

Prompts:

Why would you prevent or control disease getting onto your farm?

Why would you prevent or control disease moving off of your farm?

*Q8. If I say biosecurity, what does it make you think of?*

Prompts:

Is there something that you picture when someone says biosecurity?

What is the word that comes into your head when you hear biosecurity?

How do you feel when someone starts talking about biosecurity?

*Q9. How do you decide which measures to implement on your farm?*

Prompts:

How do you decide what to do on your farm?

What would make you decide to just drop all biosecurity measures?

What would make you take up a “new” biosecurity measure?

*Q10. If someone came to you for advice on biosecurity, what would you tell them?*

Prompts:

If I say biosecurity, who are the people you think of?

Who might want to talk to you about biosecurity?

If another farmer came to you for advice on biosecurity, what would you tell them?

*Q11. What would be your ideal biosecurity measure?*

Prompts:

What would it do?

How would it be used?

So, is biosecurity something you think you are really able to do?

*Q12. Is biosecurity really worth it?*

*Q13. Is there anything you think I might have missed, or would like to add?*

*Q14. Would you like to receive a summary of the study results? How would you like to be contacted?*

*Q15. Would you be happy to be contacted by the researchers of this team for future studies within this project? How would you like to be contacted?*

*Q16. Would you like to be entered into the prize draw for £100 worth of vouchers for a store of your choice? How would you like to be contacted?*
